# The complete chloroplast genome of *Primula calliantha* subsp. *bryophila*, an ornamental alpine plant from China

**DOI:** 10.1080/23802359.2022.2150066

**Published:** 2023-01-01

**Authors:** Rui Li, Li Zhang, Yunqi Liu, Shubao Wang, Yuan Huang

**Affiliations:** School of Life Sciences, Yunnan Normal University, Kunming, P. R. China

**Keywords:** Chloroplast genome, *Primula calliantha* subsp. *bryophila*, phylogenomic analysis

## Abstract

*Primula calliantha* subsp. *bryophila* (Balf. f. et Farrer) W.W. Smith and Forrest (1928) is a perennial alpine species with ornamental value. It is distributed in northwestern Yunnan and adjacent eastern Tibet of China, and northern Myanmar. Here, we sequenced and assembled complete plastid genome of *P. calliantha* subsp. *bryophila*, which is a circular molecule of 152,045 bp in length, including a large single-copy region (83,966 bp), a small single-copy region (17,663 bp), and a pair of inverted repeats (25,208 bp). The chloroplast genome contained 113 genes, including 79 protein-coding genes, four rRNA genes, and 30 tRNA genes. The phylogenetic tree based on chloroplast genomes showed the relative relationship of *P. calliantha* subsp. *bryophila* and *P. calliantha*, which further supports *P. calliantha* subsp. *bryophila* as a subspecies of *P. calliantha* in taxonomy. The complete chloroplast (cp) genome of *P. calliantha* subsp. *bryophila* provides valuable data for further phylogenetic studies of Primulaceae.

*Primula calliantha* subsp. *bryophila* is a perennial alpine species in the Sect. *Crystallophlomis* Rupr. of the family Primulaceae. *P. calliantha* subsp. *bryophila* is a subspecies of *P. calliantha*, the whole chloroplast genome of *P. calliantha* (NC_034678) and its phylogenetic relationship with related species have been reported (Yang et al. 2021). The main difference from the original variant is that the calyx is shorter and the corolla tube is narrower. *P. calliantha* subsp. *bryophila* distribute in northwestern Yunnan and adjacent eastern Tibet, growing in alpine grasslands and rhododendron bushes at an altitude of 3800–4500 meters. It is also distributed in northern Myanmar (Hu Q and Kelso 1996). In this research, we first report the complete chloroplast genome sequence of *P. calliantha* subsp. *bryophila* and constructed a phylogenetic tree, which provided a scientific basis for understanding its taxonomic status.

In this research, the fresh leaves and specimen of *P. calliantha* subsp. *bryophila* were collected from Zhongdian Tianchi, Yunnan, China (N 27°37′, E 99°38′). The specimen was deposited in the Herbarium of Yunnan Normal University under the voucher number of HY20 (Kunming, China; Jianlin Hang, hjlyuun@163.com). Total genomic DNA was extracted using a modified CTAB (cetyl trimethylammonium bromide) method (Sahu et al. 2012). The fragmented genomic DNA was used to construct short-insert libraries for Illumina paired-end (PE) sequencing on the Illumina Hiseq X Ten sequencer. The clean reads were assembled by NOVOPlasty v2.7.2, and two alternative chloroplast sequences in opposite directions were obtained (Dierckxsens et al. 2016). Then the cp genome was annotated using Geneious v8.0.2 software based on the reference of *P. calliantha* (MZ054238) (Matthew et al. 2012).

The raw reads (19,100,898) were subjected to assembly to produce a circular molecule of complete chloroplast with about an average 831.8 coverage. The cp genome of *P. calliantha* subsp. *bryophila* (GenBank accession ON416873) is 152,045 bp in length, including a large single-copy region (83,966 bp), a small single-copy region (17,663 bp), and a pair of inverted repeats (25,208 bp). The overall GC content is 37%. In total, the cp genome was annotated with 113 genes, including 79 protein-coding genes, 30 tRNA genes and four rRNA genes.

In order to explore the phylogenetic relationship of *P. calliantha* subsp. *bryophila* within the genus *Primula*, 43 *Primula* cp genomes were downloaded as well as three *Androsace* species, two *Lysimachia* species and *Glaux maritima* (Liu et al. 2021) as outgroups from GenBank. These genome sequences were aligned by MAFFT software (Katoh and Standley 2013), and then a maximum likelihood phylogenetic tree was constructed using IQ_TREE v1.6.12 (Nguyen et al. 2015). The branch support was tested with 10,000 replicates using the SH-like approximate likelihood ratio (SHAlrt) (Guindon et al. 2010) and ultrafast bootstrap approximation (UFboot) (Hoang et al. 2018). The optimal model according to the Bayesian information criterion was TVM + F+R3 using ModelFinder (Kalyaanamoorthy et al. 2017) and used FigTree v1.4.4 software to further beautify the phylogenetic tree (Figure 1). The phylogenetic tree based on chloroplast genomes showed the relative relationship of *Primula calliantha* subsp. *bryophila* and *P. calliantha*, which further supports *P. calliantha* subsp. *bryophila* as a subspecies of *P. calliantha* in taxonomy (Figure 2). The complete chloroplast genome of *P. calliantha* subsp. *bryophila* provides valuable data for further phylogenetic studies of Primulaceae (Figure 3).

**Figure 1.**
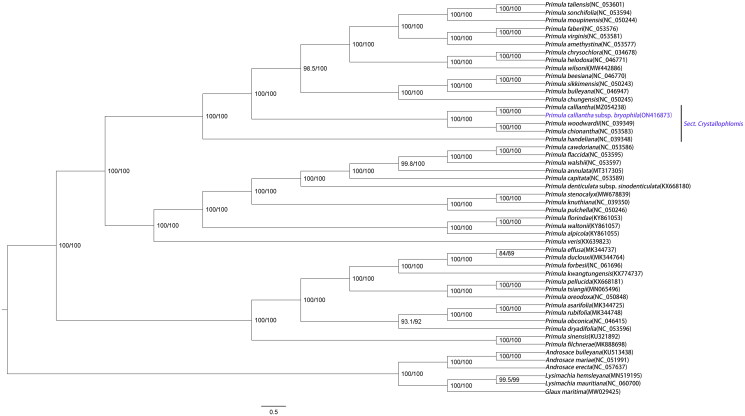
ML phylogenetic tree of *Primula calliantha* subsp. *bryophila* and 49 Primulaceae species based on complete chloroplast genome, branch supports values were reported as SH-aLRT/UFBoot.

**Figure 2.**
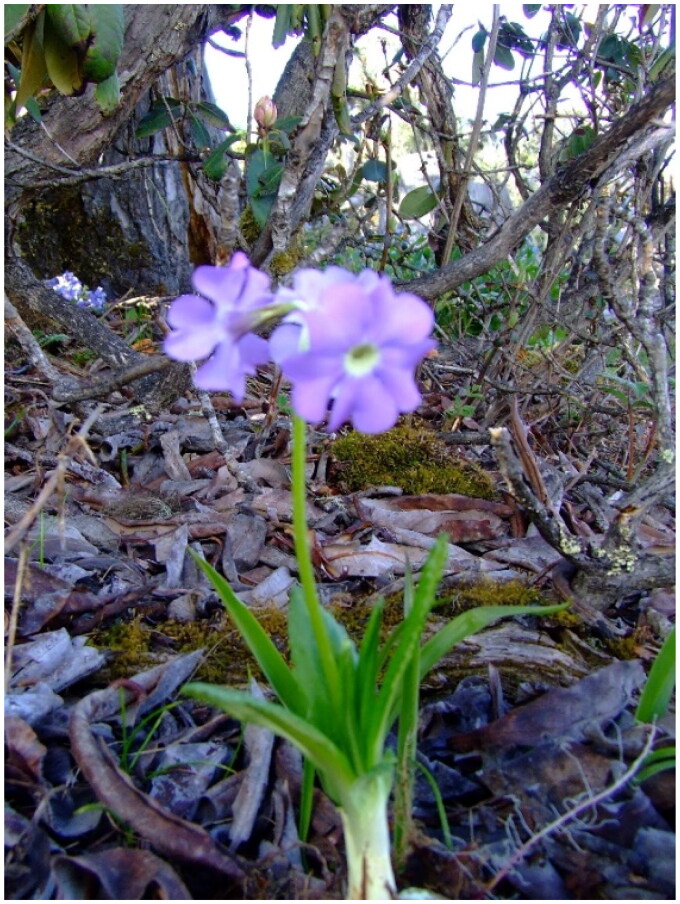
Species reference map of *Primula calliantha* subsp. *bryophila* (Species reference map was taken by Yuan Huang in Zhongdian Tianchi, Yunnan, China (N 27°37′, E 99°38′)).

**Figure 3.**
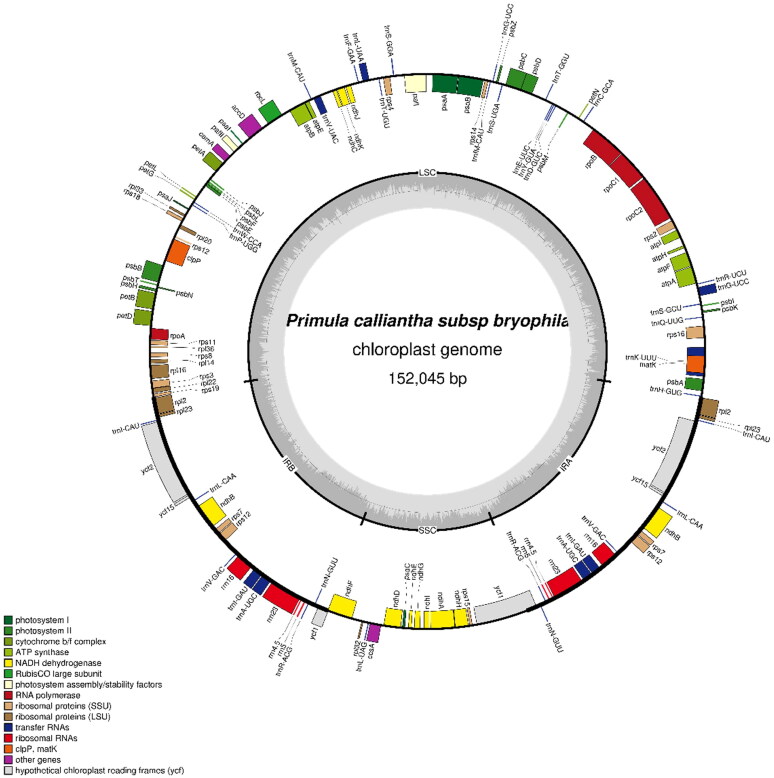
Chloroplast gene map of *Primula calliantha* subsp. *bryophila.*

## Data Availability

The genome sequence data that support the findings of this study are openly available in GenBank of NCBI at [https://www.ncbi.nlm.nih.gov/] under the accession no. ON416873. The associated BioProject, SRA, and Bio-Sample numbers are PRJNA834972, SRR19141994, and SAMN28088249, respectively.
